# Supplementary material for: Testing times: disentangling admixture histories in recent and complex demographies using ancient DNA
Source: Genetics. 2024 Jul 16;228(1):iyae110. doi: 10.1093/genetics/iyae110 (PMC11373510; doi:10.1093/genetics/iyae110)
Supplement: iyae110_Supplementary_Data [file iyae110_supplementary_data.zip › Supplemental_Material_GENETICS-2024-307001.docx]

**SUPPLEMENTARY MATERIAL**

**Testing Times: Disentangling Admixture Histories in Recent and Complex Demographies using ancient DNA**

Matthew P. Williams^1*^, Pavel Flegontov^2,3^, Robert Maier^3^, Christian D. Huber^1*^

1: Department of Biology, The Pennsylvania State University, University Park, PA 16802, USA
2: Department of Biology and Ecology, University of Ostrava, Ostrava 701 03, Czechia
3: Department of Human Evolutionary Biology, Harvard University, Cambridge, MA 02138, USA

^*^Correspondence to Matthew. P. Williams: mkw5910@psu.edu, and Christian D. Huber: cdh5313@psu.edu

### Figure S1


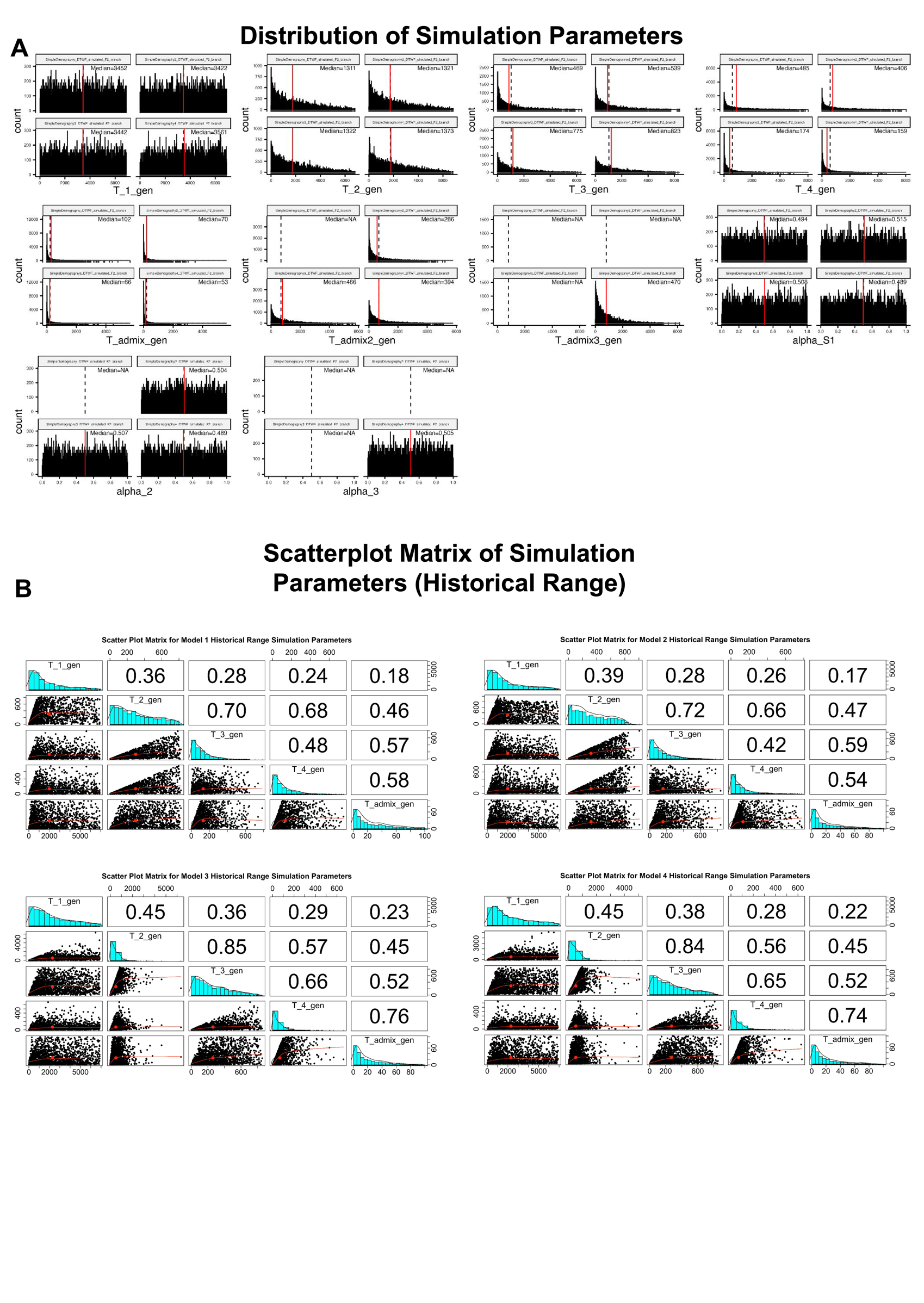


Figure S1: The distributions of simulation parameters for the simple demographic Models shown in the Main publication Figure 3A-D. For each simulation parameter the Models are ordered; top left (Model 1), top right (Model 2), bottom left (Model 3), and bottom right (Model 4). The median value for each simulation parameter within each demographic Model is printed in the top right corner of each box. The median value across all demographic Models for each simulation parameter is represented with the red dotted line. (A) The unconstrained distributions for each of the simulation parameters (T1, T2, T3, T4, Tadmix, Tadmix2, and Tadmix3, alpha_S1, alpha_2, alpha_3). Note that in Model 2, Tadmix2 is the admixture event from the outgroup R3 branch to the S1 population and alpha_2 is its simulated weight parameter. In Model 3, Tadmix2 is the admixture event from the S2R2 branch to S1 and alpha_2 is its simulated weight parameter. The parameter Tadmix 3 is the additional admixture parameter in Model 4 and corresponds to admixture from the S2R2 branch to S1 and alpha_3 is its simulated weight parameter. (B) Scatterplot matrix of simulation parameters (T1, T2, T3, T4, Tadmix) for each demographic Model under the constraint of median pairwise *F_ST_* (S1, S2, R1, R2, and R3 populations) between 0 and 0.02 and Tadmix less than or equal to 100 generations. The plots were generated with R package psych using the Spearman correlation method.

### Figure S2


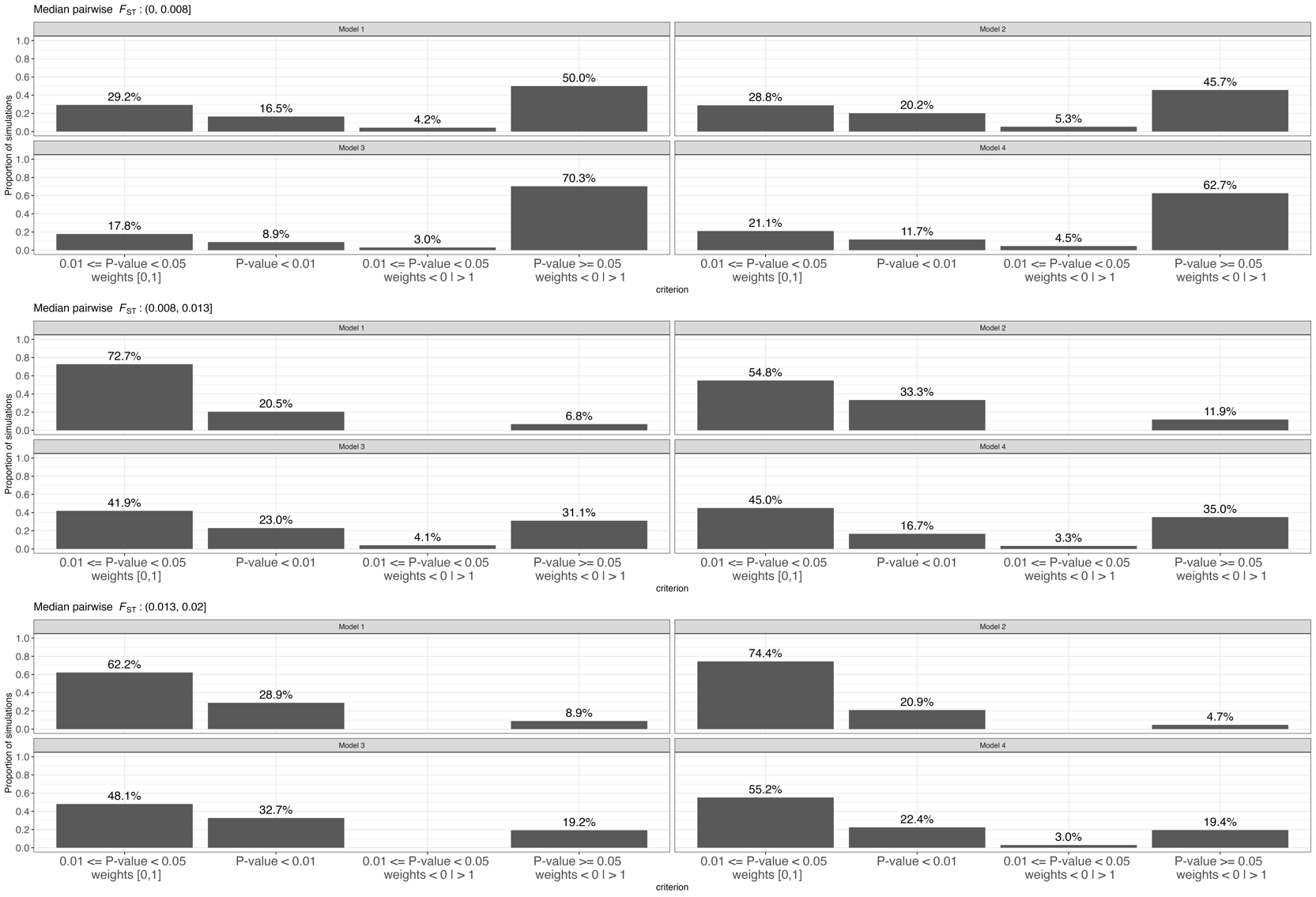


Figure S2: The frequency of conditions leading to the rejection of the true qpAdm model (S1+S2) under varying *F*_ST_ ranges. The Y-axis shows the frequency of that condition across the replicates and the number above each barplot shows the proportion of the total conditions that each makes up.

### Figure S3


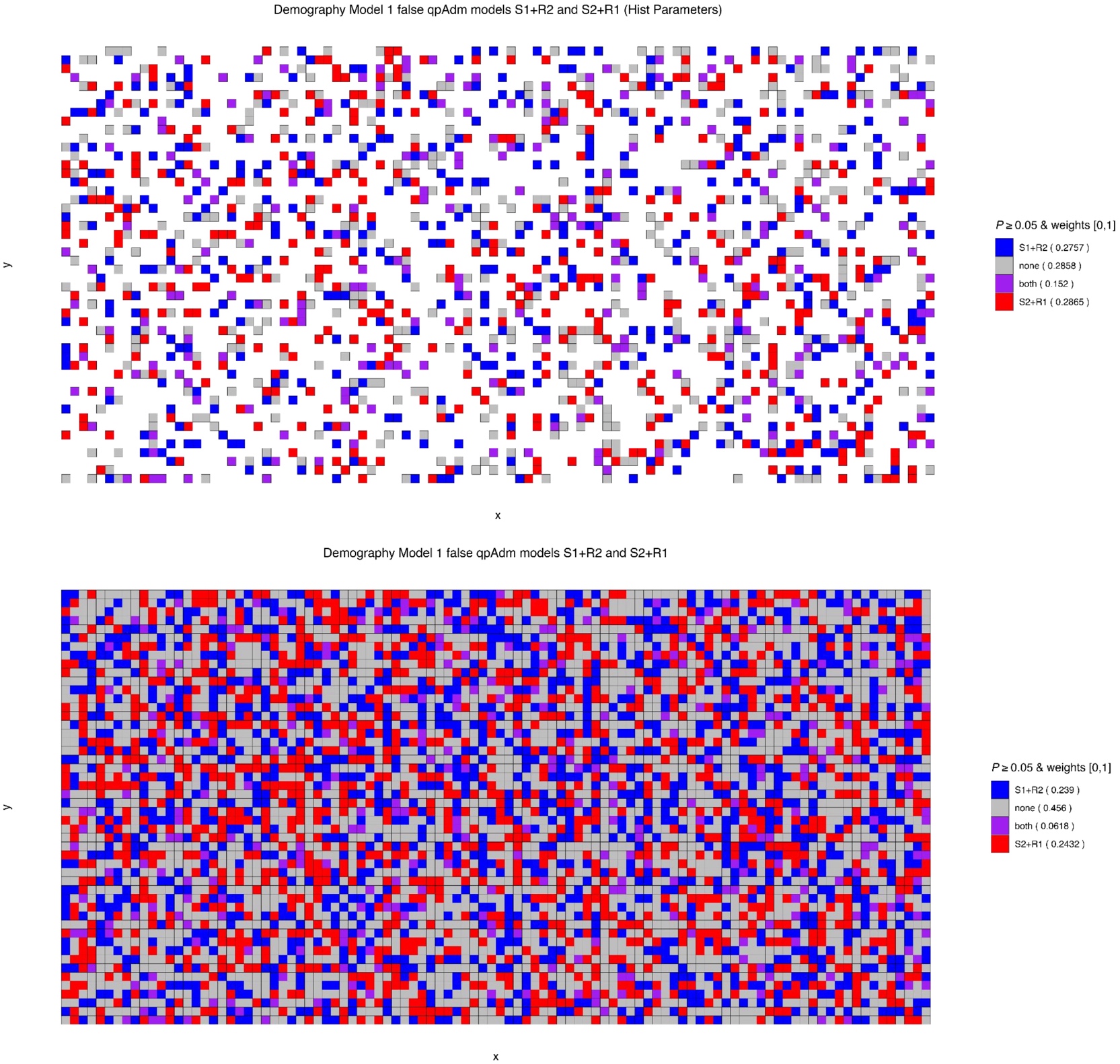


Figure S3: Dot matrix plot showing for the historical conditions (top) and full parameter range (bottom) for the cases where in each replicate both false models (S1+R2 & S2+R1) are plausible (purple), or only one of the false models is plausible (red or blue), or both false models are rejected (white).

### Figure S4


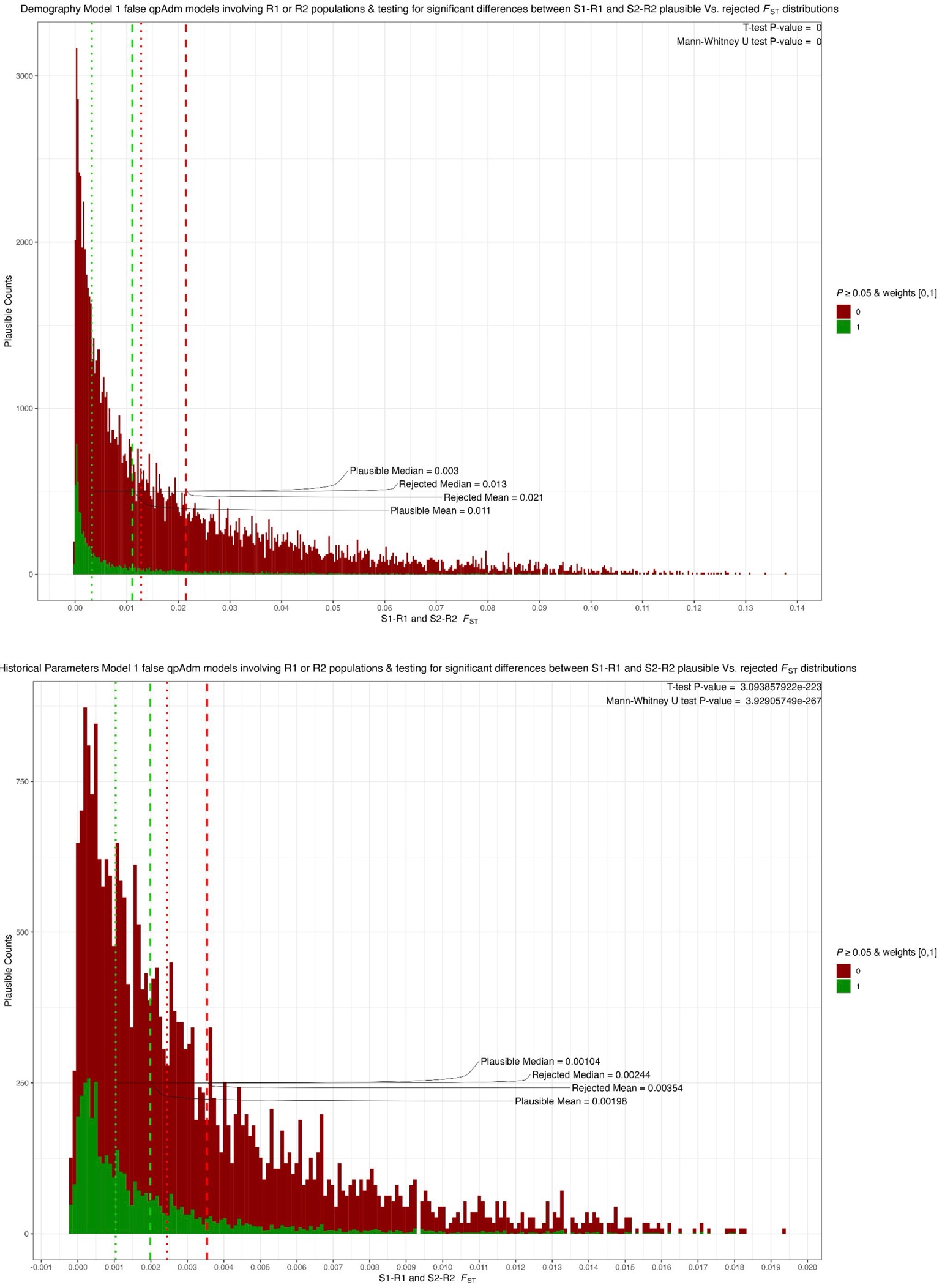


Figure S4: The joint distribution of *F*_ST_ (S1-R1 and S2-R2) for the false qpAdm models involving one of the R1 or R2 populations (Model 1). False models that are plausible have smaller *F*_ST_ between the clades of S1-R1 and S2-R2.

### Figure S5


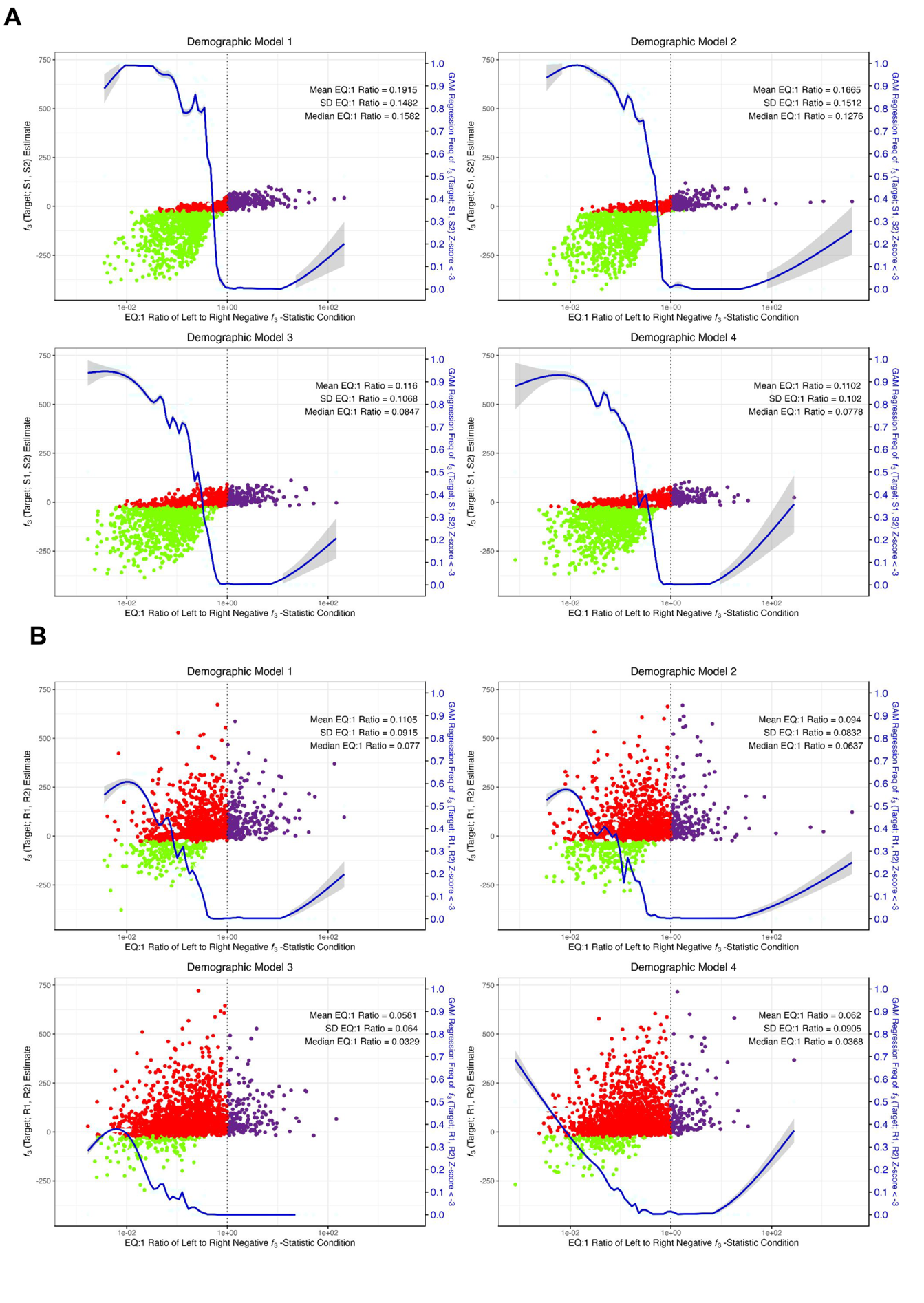


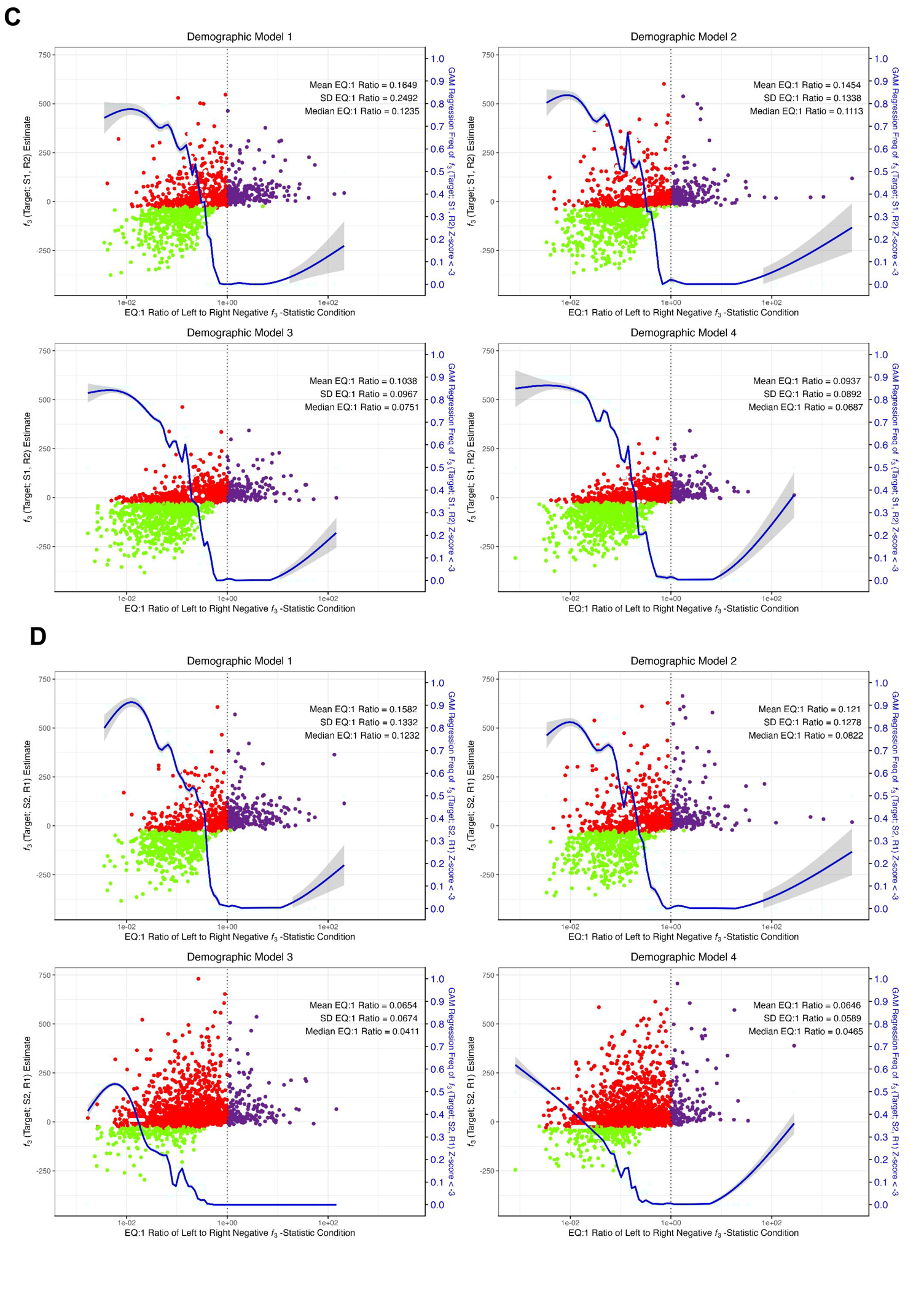


Figure S5: The *f*_3_-statistic condition, EQ:1 from the manuscript where we are dividing the left side of the condition by the right side (2 alpha (1 - alpha)). Each plot shows on the x axis the EQ:1 *f*_3_-statistic condition and on the y axis the *f*_3_-statistic estimate. Green points show the *f*_3_-statistic estimate with a Z-score < -3, purple points show the *f*_3_-statistic estimate with a Z-score > -3, and red points are those with an EQ:1 *f*_3_-statistic negativity condition < 1 (i.e., LHS < RHS), but the *f*_3_-statistic estimate has a Z-score > -3 and thus not significant (i.e., false negative). In each top right corner of each plot is shown the mean, standard deviation, and median of the EQ:1 negative *f*_3_-statistic condition for significant *f*_3_-statistics (Z-scores <-3). (A) the *f*_3_(Target; S1, S2), i.e., the “true model”. “False models” shown in (B) *f*_3_(Target; R1, R2), (C) *f*_3_(Target; S1, R2), and (D) *f*_3_(Target; S2, R1).

### Figure S6


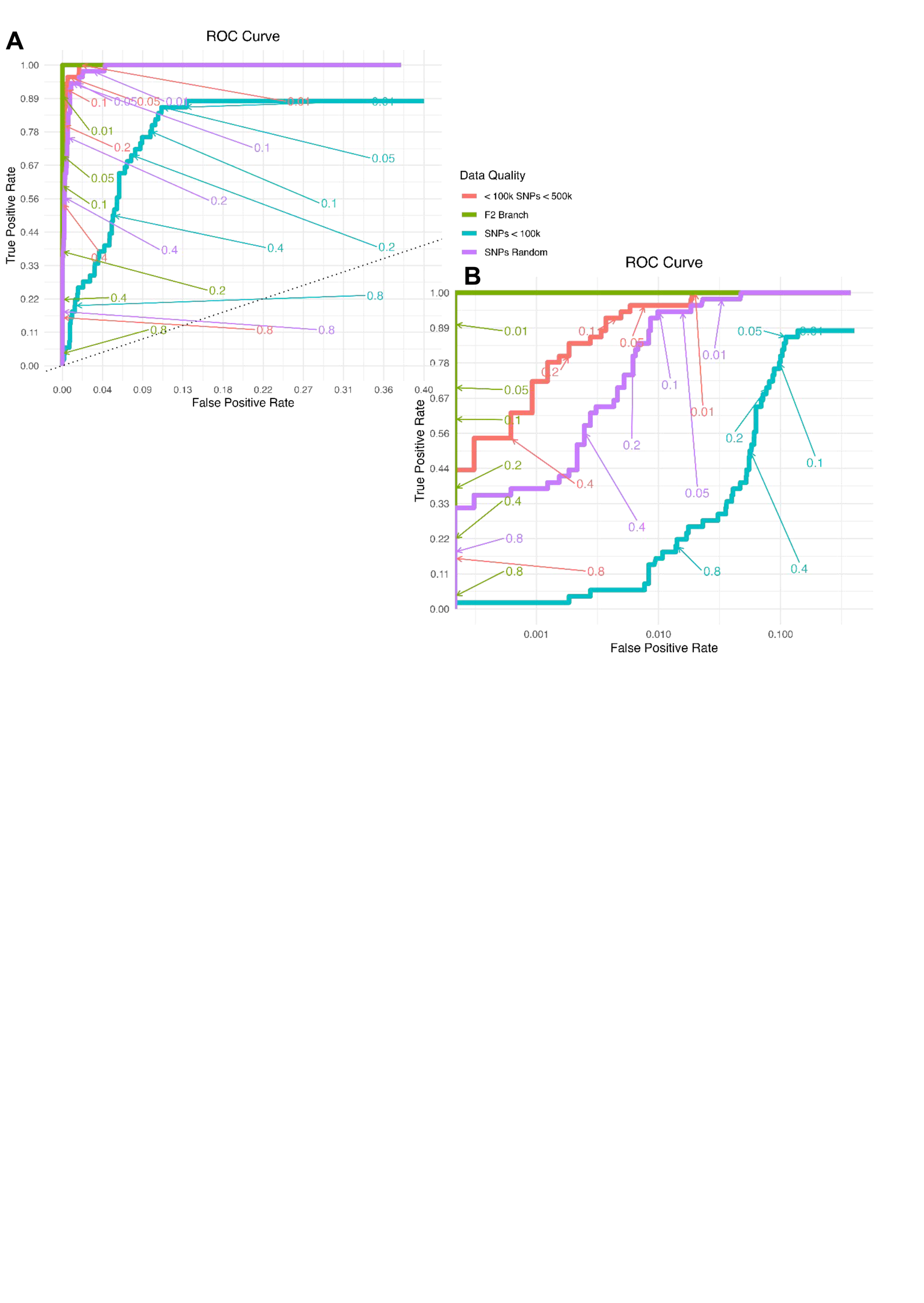


Figure S6: Receiver operating characteristic curve (ROC) analysis on *P*-values. Each ROC analysis was computed on 3,000 *P*-values between zero and one. The True Positive Rate is the number of plausible (*P*-value greater than or equal to the iterator value) true qpAdm model observations (TP) divided by the sum of the number of TP and the number of times the true model is rejected (FN) (*P*-value less than the iterator value). The False Positive Rate was calculated similarly, with the number of FP models divided by the sum of FP and true rejections (TN) of single and two-source false qpAdm models. Colors represent different levels of missing data conditions. (A) the ROC analysis across the entire FP range. (B) ROC analysis with the FP range on a log10 scale.

### Figure S7


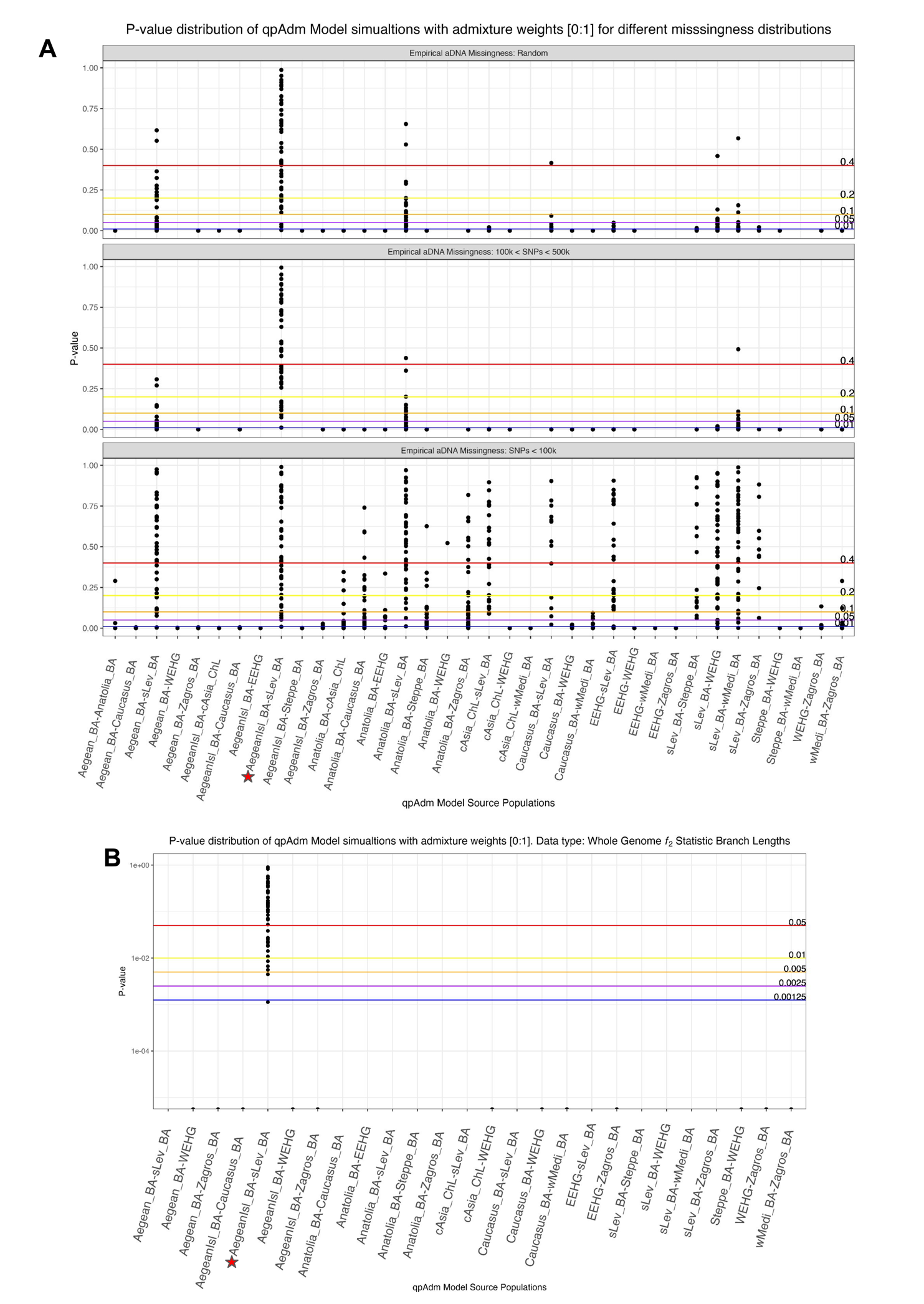

Figure S7: Distribution of *P*-values for qpAdm models with admixture weights [0:1]. (A) Distribution of *P*-values for different amounts of data missingness. Horizontal lines show various *P*-values thresholds in the bottom to top order: blue = 0.01, purple = 0.05, orange = 0.1, yellow = 0.2, and red = 0.4. (B) Distribution of *P*-values for the *f_2_* branch data condition. Horizontal lines show various *P*-value thresholds bottom to top order: blue = 0.00125, purple = 0.0025, orange = 0.005, yellow = 0.01, and red = 0.05. Red star below the x axis qpAdm models shows the true model.

### Figure S8


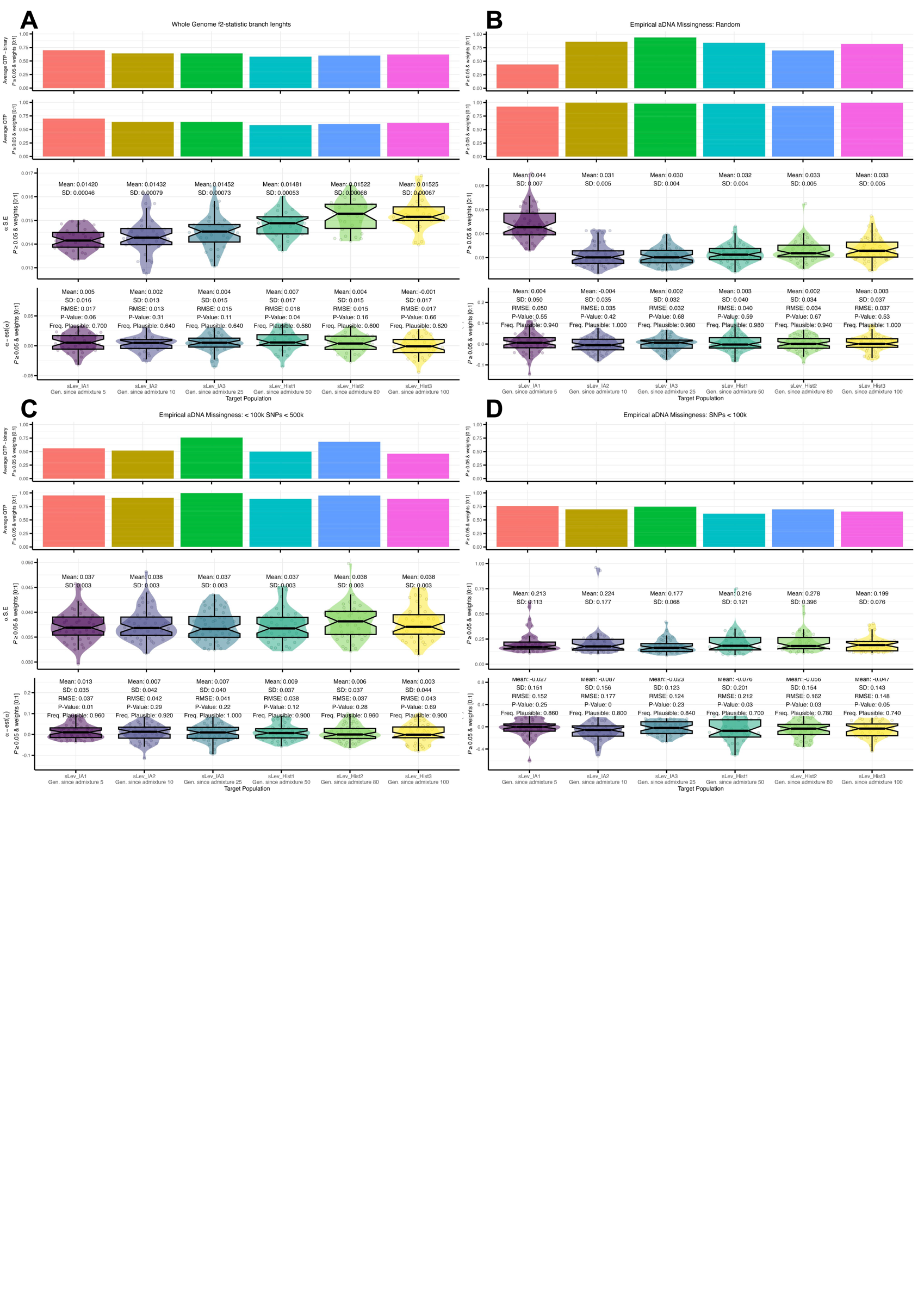


Figure S8: Generations since admixture and qpAdm performance (QTP). Measures of qpAdm performance (QTP-binary (top row) and QTP (second row)) averaged across all 50 simulations. The accuracy and precision of qpAdm admixture weight estimates is measured through the average and SD of the estimated standard error across all simulations (middle row) and the average, SD, root-mean-standard error, one-sample t-test *P*-value of the delta-alpha calculation (bottom row). The frequency across all replicates of the plausible true qpAdm model is written at the bottom. (A) Results computed on the branch-length *f_2_*-statistic data. (B) Computed on the random sampling of empirical aDNA data missingness (C) Computed on the random sampling of empirical aDNA data missingness constrained to have samples with 100k to 500k SNPs covered. (D) Computed on the random sampling of empirical aDNA data missingness constrained to have samples with less than 100k SNPs covered.

###

### Figure S9


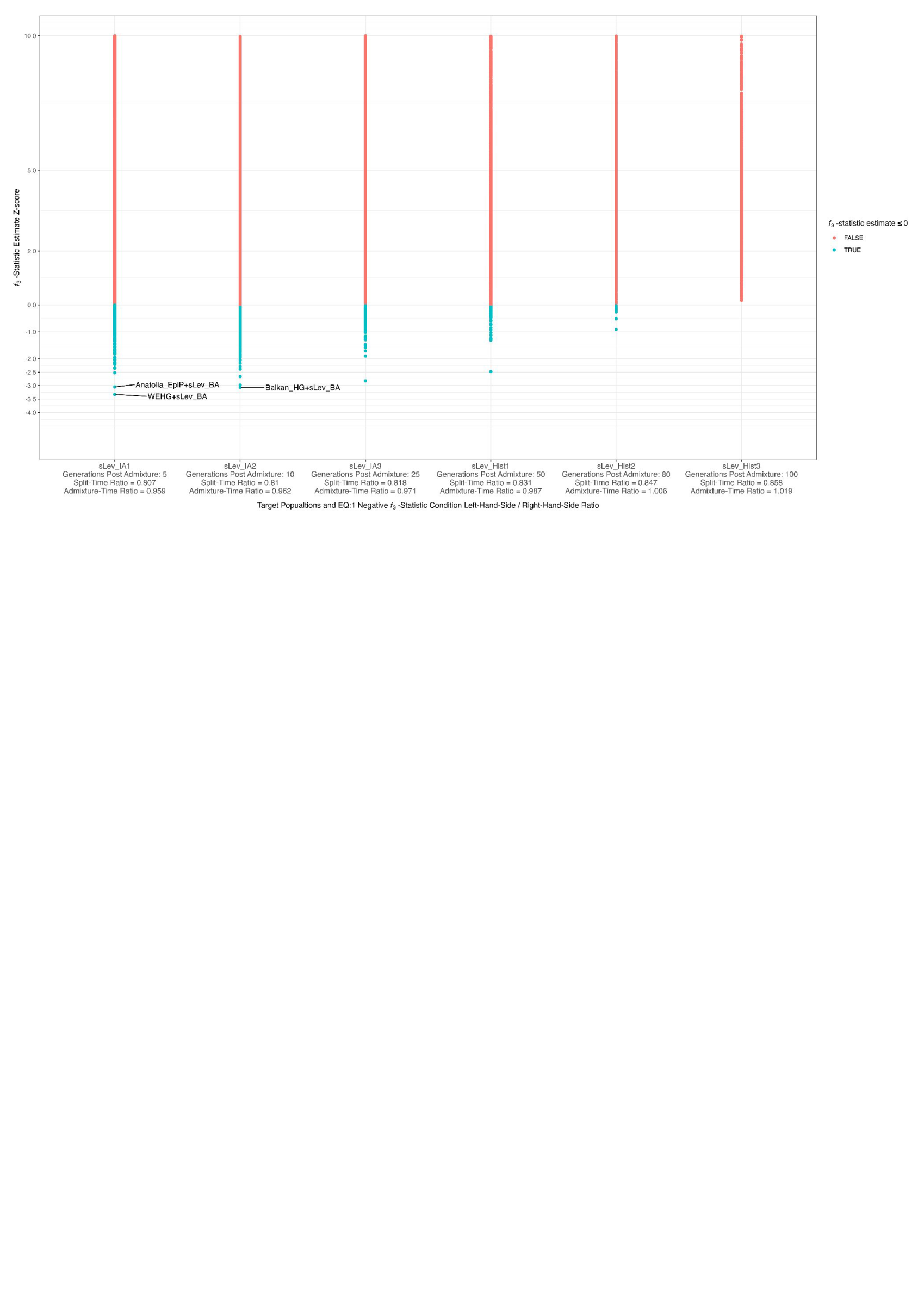


Figure S9: The Z-score for the *f_3_*-statistic estimate computed for the six Target populations each with increasing generations since admixture. *f_3_*-statistic values less than zero are green. Two values are printed below each Target population on the x-axis. The Split-Time Ratio is the *f*_3_-statistic negativity condition, EQ:1 from the manuscript where we are dividing the left side of the condition by the right side (2 alpha (1 - alpha)). The Tadmix parameter for the Split-time Ratio is computed using the split age-parameter of the Levant and Aegean populations. The Admixture-Time Ratio uses the generation of the most recent bi-directional gene-flow between the Levant and Aegean lineages as the value for the Tadmix parameter in calculating the *f*_3_-statistic negativity condition, EQ:1.

### Figure S10


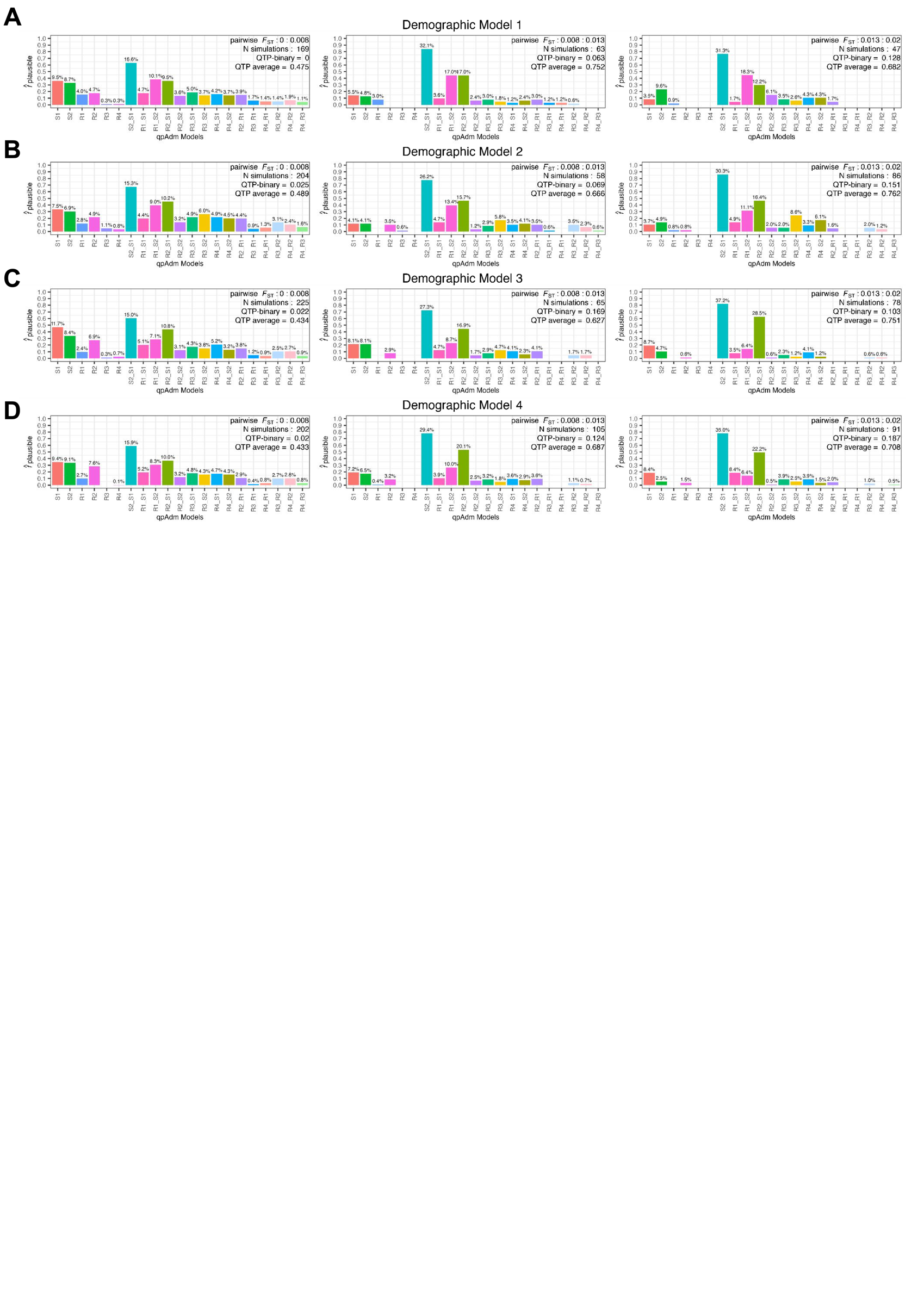


Figure S10. Increase in plausible single-source qpAdm models with admixture weights near the boundaries. Barplots of plausible *qpAdm* models conditional on parameter space of the simulated admixture weight < 0.1 or > 0.9, median pairwise *F_ST_* between the S1, S2, R1, R2, and R3 populations between 0 and 0.02, and the number of generations since admixture less than 100. Each row represents one simulated demographic history and the columns are increasing ranges of population differentiation (*F_ST_*) corresponding to the historical period demarcations indicated in Figure 1 B.

### Table S1


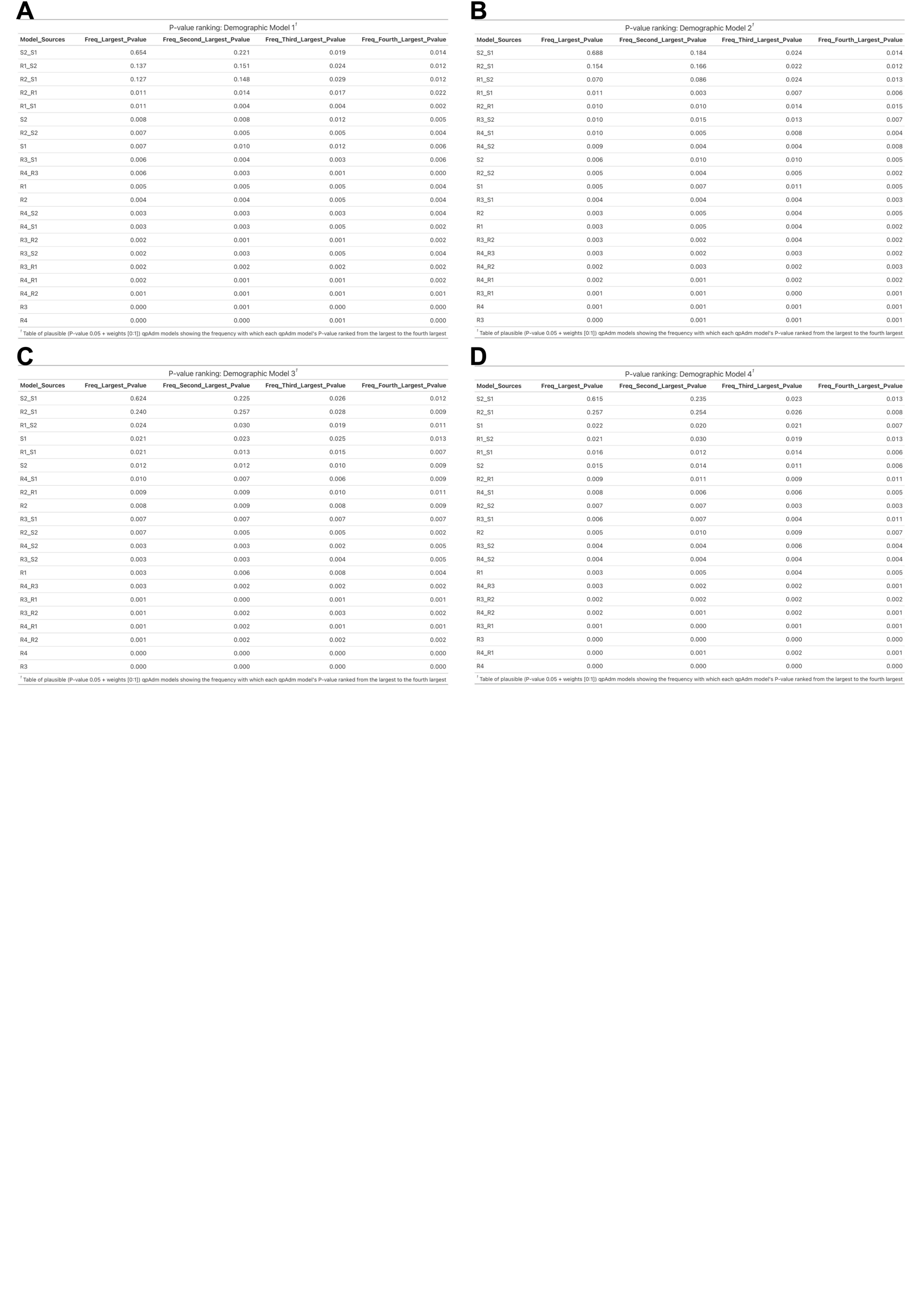


Table S1: Relative ranking of plausible qpAdm models by *P*-value for the four simple demographic Models.

### Table S2

| *Simulated Data Missingness* | | | | |
| --- | --- | --- | --- | --- |
| pop | mu_missing | max_missing | min_missing | sd_missing |
| sLev_IA1 | 0.89 | 0.95 | 0.76 | 0.05 |
| sLev_IA2 | 0.51 | 0.89 | 0.32 | 0.14 |
| sLev_IA3 | 0.49 | 0.69 | 0.36 | 0.08 |
| sLev_Hist1 | 0.58 | 0.78 | 0.38 | 0.09 |
| sLev_Hist2 | 0.63 | 0.76 | 0.47 | 0.08 |
| sLev_Hist3 | 0.60 | 0.91 | 0.35 | 0.12 |
| ascertain_CEU | 0.00 | 0.00 | 0.00 | 0.00 |
| ascertain_CHB | 0.00 | 0.00 | 0.00 | 0.00 |
| ascertain_AFR | 0.00 | 0.00 | 0.00 | 0.00 |
| ascertain_sAs | 0.00 | 0.00 | 0.00 | 0.00 |
| Mbuti | 0.00 | 0.00 | 0.00 | 0.00 |
| UstIshim | 0.63 | 0.86 | 0.39 | 0.10 |
| nEur_HG | 0.73 | 0.92 | 0.44 | 0.10 |
| nAfr_EpiP | 0.78 | 0.92 | 0.54 | 0.09 |
| sLev_EpiP | 0.76 | 0.91 | 0.52 | 0.09 |
| Zagros_Neo | 0.67 | 0.87 | 0.43 | 0.10 |
| Balkan_HG | 0.72 | 0.92 | 0.53 | 0.09 |
| Caucasus_HG | 0.73 | 0.89 | 0.54 | 0.08 |
| Anatolia_EpiP | 0.73 | 0.93 | 0.56 | 0.08 |
| sLevant_Neo | 0.78 | 0.99 | 0.55 | 0.12 |
| Caucasus_BA | 0.86 | 0.99 | 0.26 | 0.13 |
| Zagros_BA | 0.86 | 0.99 | 0.36 | 0.15 |
| cAsia_ChL | 0.65 | 0.99 | 0.31 | 0.21 |
| wMedi_BA | 0.45 | 0.81 | 0.27 | 0.12 |
| sLev_BA | 0.51 | 0.81 | 0.28 | 0.14 |
| Aegean_BA | 0.64 | 0.90 | 0.38 | 0.14 |
| AegeanIsl_BA | 0.69 | 0.91 | 0.40 | 0.12 |
| Steppe_BA | 0.64 | 0.92 | 0.44 | 0.13 |
| WEHG | 0.55 | 0.86 | 0.33 | 0.11 |
| EEHG | 0.49 | 0.72 | 0.27 | 0.10 |
| Anatolia_BA | 0.46 | 0.66 | 0.28 | 0.09 |
| BasalEurasian | 0.52 | 0.65 | 0.29 | 0.08 |
| eEurasia | 0.54 | 0.74 | 0.36 | 0.08 |
| nEurasia | 0.54 | 0.86 | 0.34 | 0.13 |
| OOA | 0.77 | 0.95 | 0.33 | 0.19 |
| NearEast | 0.73 | 0.95 | 0.33 | 0.19 |
| eNearEast | 0.68 | 0.96 | 0.08 | 0.19 |
| wNearEast | 0.85 | 0.99 | 0.42 | 0.13 |
| nwNearEast | 0.53 | 0.77 | 0.35 | 0.10 |
| *Note. Missingness from the random sampling scheme of Southwest Asian individuals from Allen Ancient DNA Resource (AADR) v.52.2.* | | | | |

### Table S3


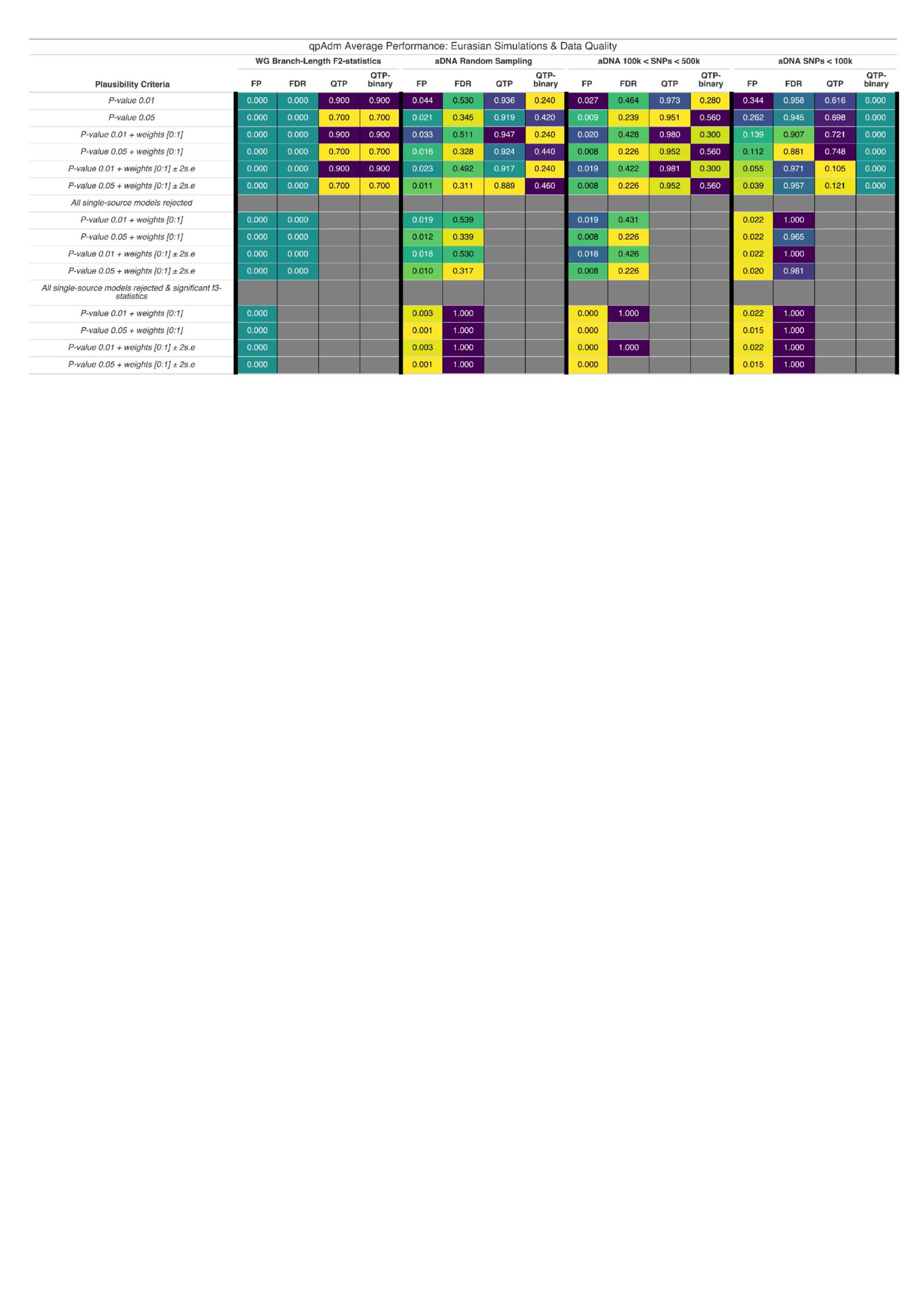


Table S3: Performance summaries of rotating qpAdm analysis on the West Eurasian simulations for the four datasets differing in data missingness levels and for different performance metrics: FP = false positive rate, FDR = false discovery rate, QTP = qpAdm test performance, QTP-binary = qpAdm test performance provided that only the true model fits the data.

### Table S4


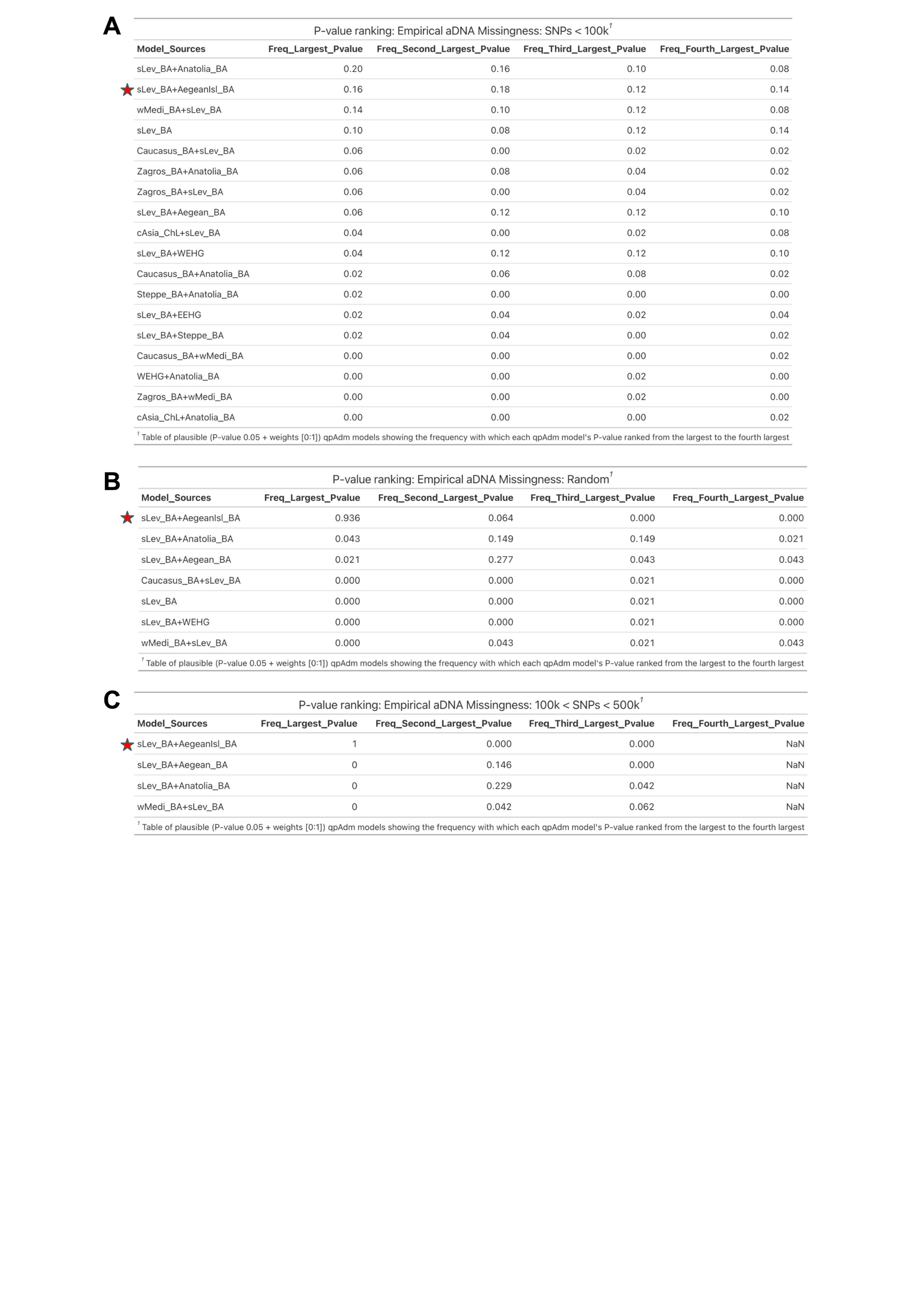


Table S4: Relative ranking of plausible qpAdm models by *P*-value. A red star is placed next to the true model. (A) Lowest-coverage aDNA dataset. (B) Random sampling of empirical missingness. (C) Constraint to individuals with 100k to 500k SNPs covered.

### Figure S11


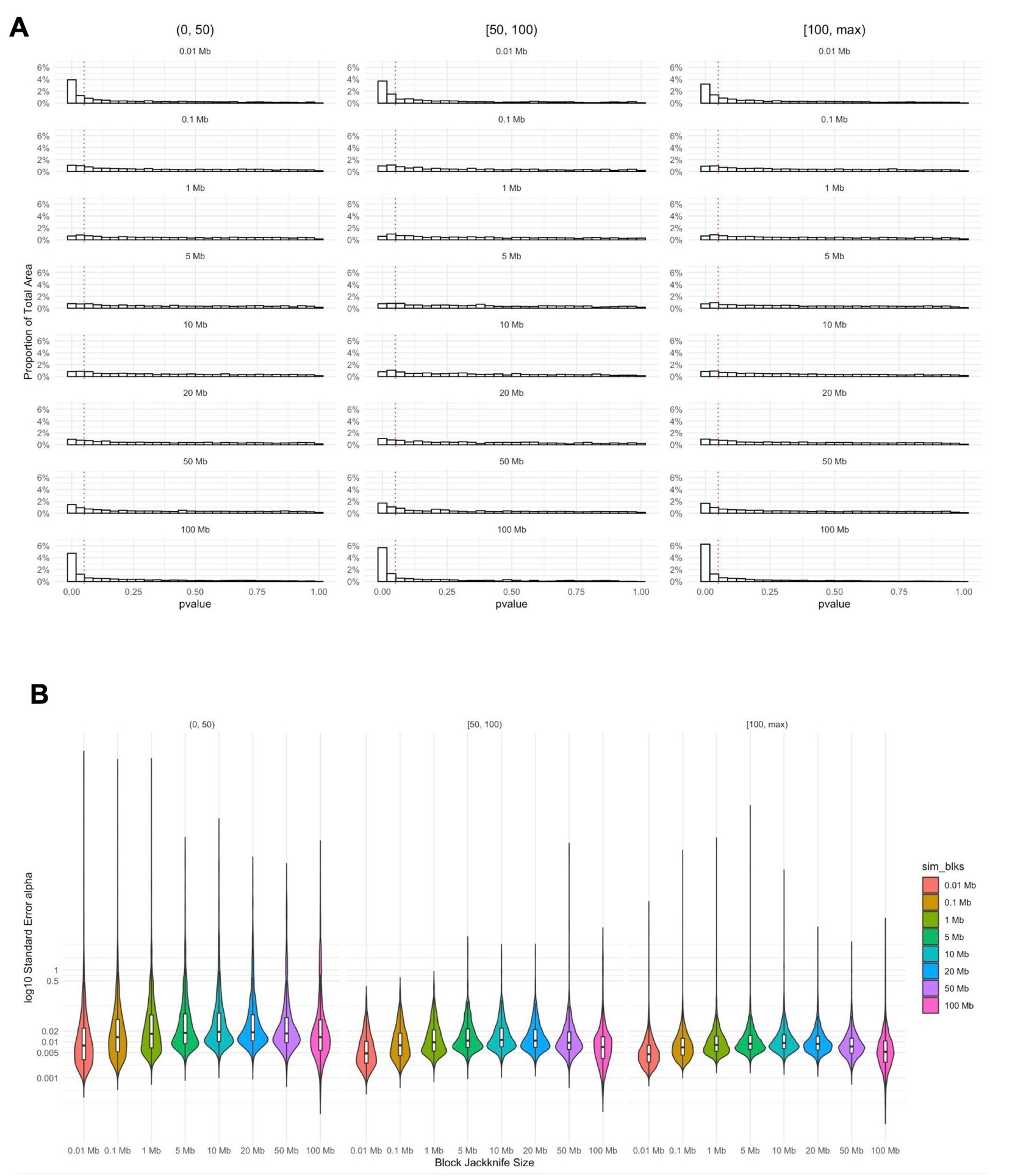


Figure S11. (A) Distribution of qpAdm P-values under simple demographic Model 1 for eight block sizes (0.01 to 100 Mb) referenced from Harney et al. and generations since admixture (T_admix_ ranges = (0, 50), [50, 100), and [100, max)). (B) Violin and boxplot plot of the qpAdm standard error under the same jackknife block sizes and generations since admixture bins.

### Figure S12

Figure S12. qpAdm weight estimate bias under simple demographic Model 1 for varying jackknife block size and generations since admixture. The bias is estimated under three temporal bins of generations since admixture (T_admix_ ranges = (0, 50), [50, 100), and [100, max)) and eight block sizes (0.01 to 100 Mb) referenced from Harney et al. The bias (delta alpha) is measured as the difference between the simulated alpha and estimated alpha values. Histogram of the delta alpha is shown in a constrained range to visually account for outliers, and a scatterplot of the entire range is included within each jackknife block size and generation bin.

### Figure S13


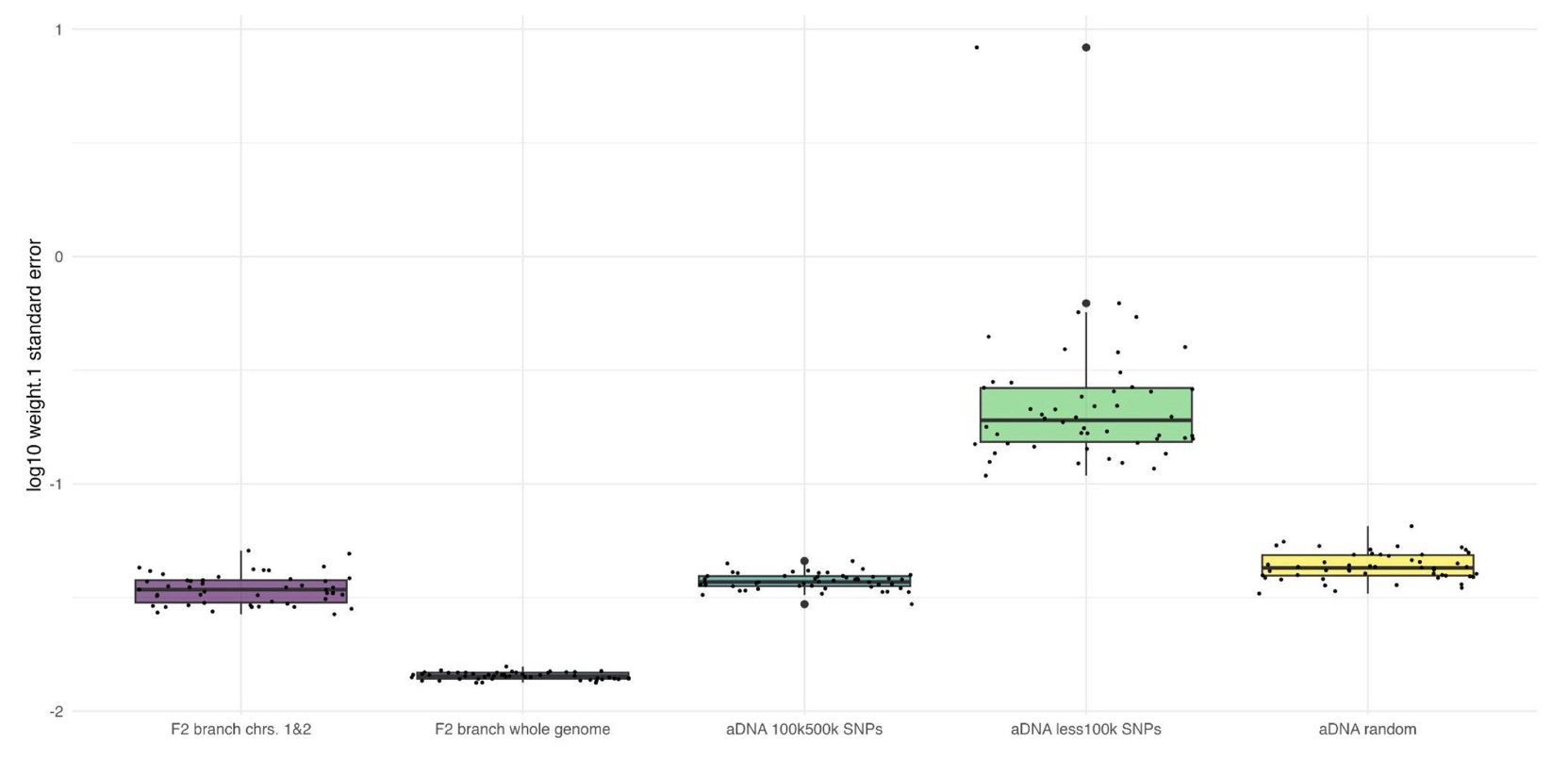


Figure S13. Evaluation of the qpAdm standard error under varying simulated genome sizes and ancient DNA data missingness conditions using the complex simulation. The *f*_2_-statistic computed on chromosomes 1 and 2 was subsampled from the whole-genome simulations and qpAdm re-run to generate standard errors.
